# Supplementary material for: A pan-cancer analysis of PBAF complex mutations and their association with immunotherapy response
Source: Nat Commun. 2020 Aug 20;11:4168. doi: 10.1038/s41467-020-17965-0 (PMC7441387; doi:10.1038/s41467-020-17965-0)
Supplement: Supplementary file 2 — Supplementary Information [file 41467_2020_17965_MOESM2_ESM.pdf]

A Pan-Cancer Analysis of PBAF Complex Mutations and Their Association with Immunotherapy Response

Hakimi et al.

Supplementary Figure 1: TCGA pan-cancer analysis of the frequency of alterations in *PBRM1, ARID2,* and *BRD7.*

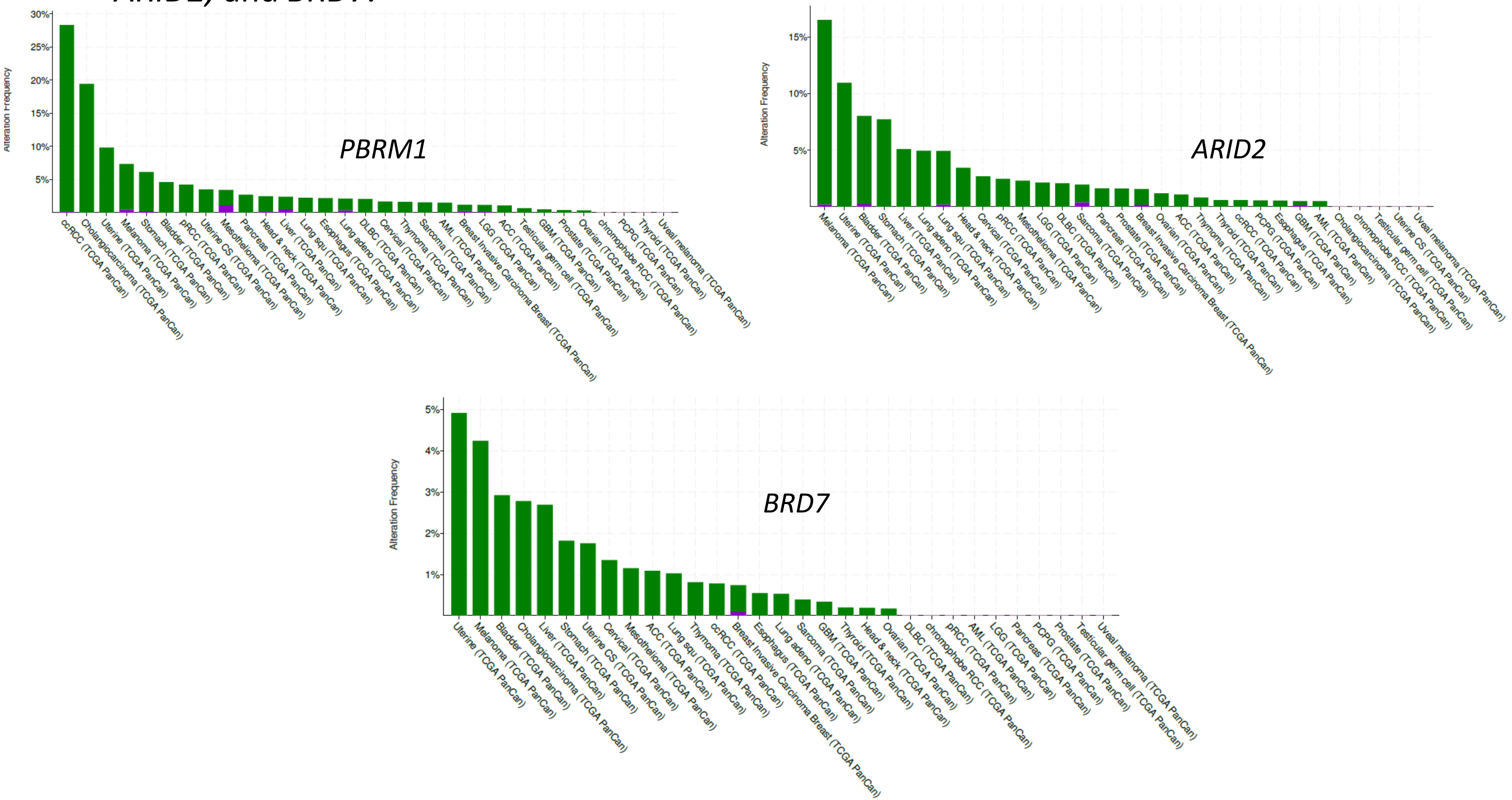

Supplementary Figure 2: MSK-IMPACT pan-cancer analysis of the frequency of alterations in *PBRM1* and *ARID2.*

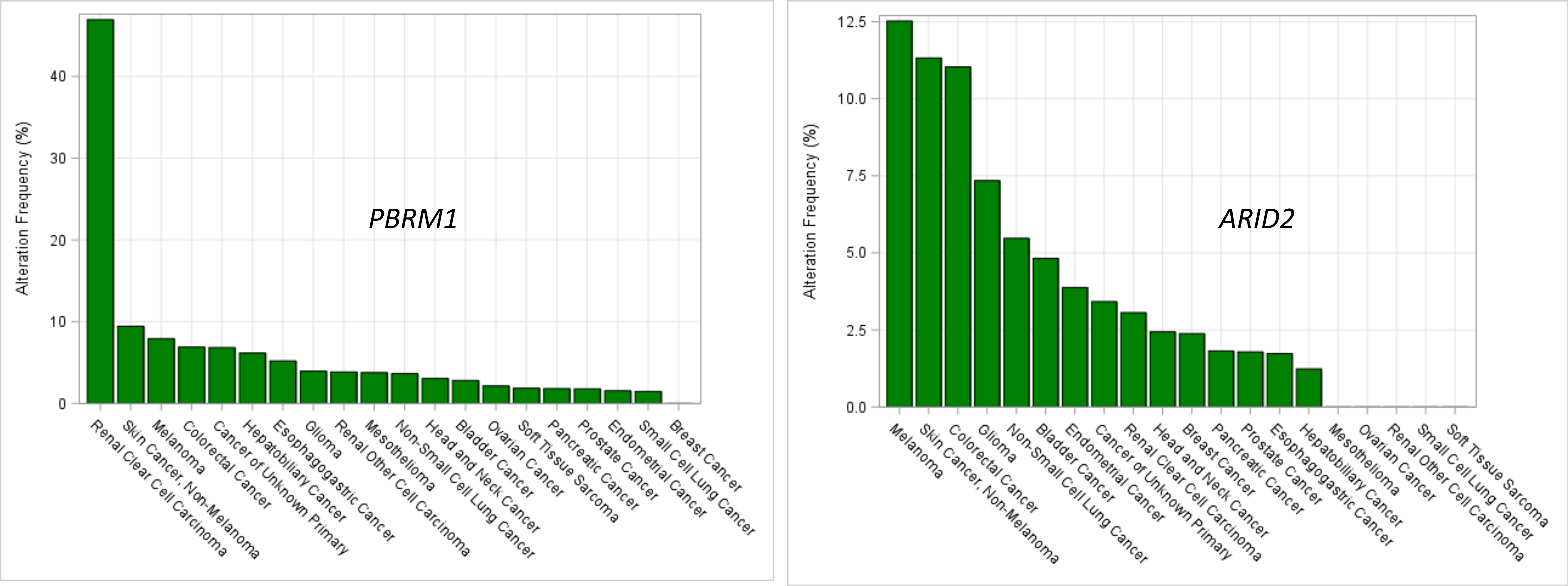

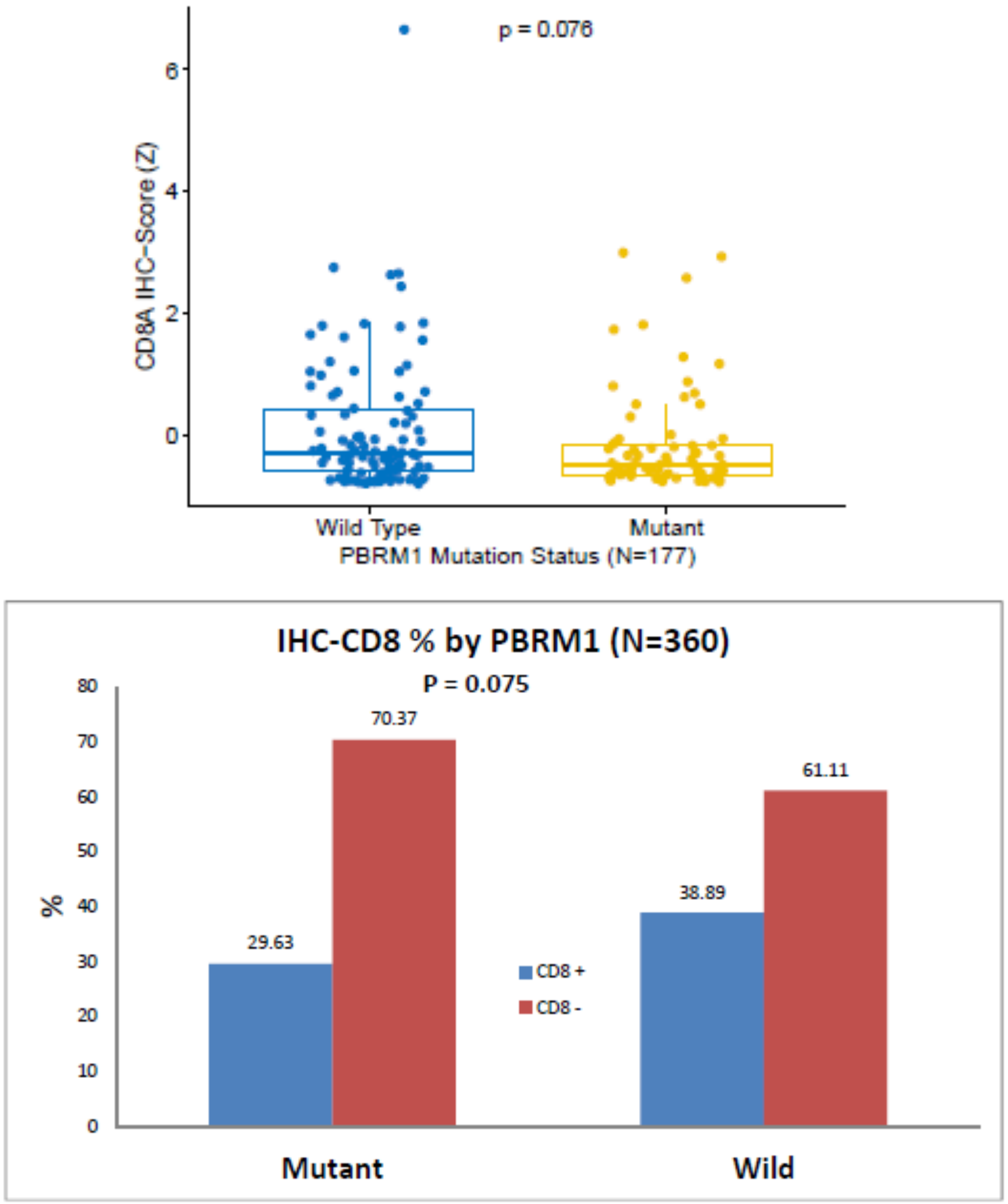

Supplementary Figure 3:
Immunohistochemistry staining results from COMPARZ[1] and McDermott et al[2] data sets demonstrate no difference in CD8 positivity between *PBRM1* mutant and wild-type tumors. Box plot: middle line of box indicates median and the bounds indicate quartile 1 and quartile 3. The whiskers reach to the maximum/minimum point within the 1.5 x interquartile range from quartile 3/quartile 1, respectively. *p*-values from COMPARZ bar plots generated by Fisher's Exact test; *p*-values from the box plots derived from Wilcoxon rank-sum test. The Fisher's Exact test and Wilcoxon rank-sum test *p*-values are two-sided. No adjustments made for multiple comparisons; all *p*-values are nominal.

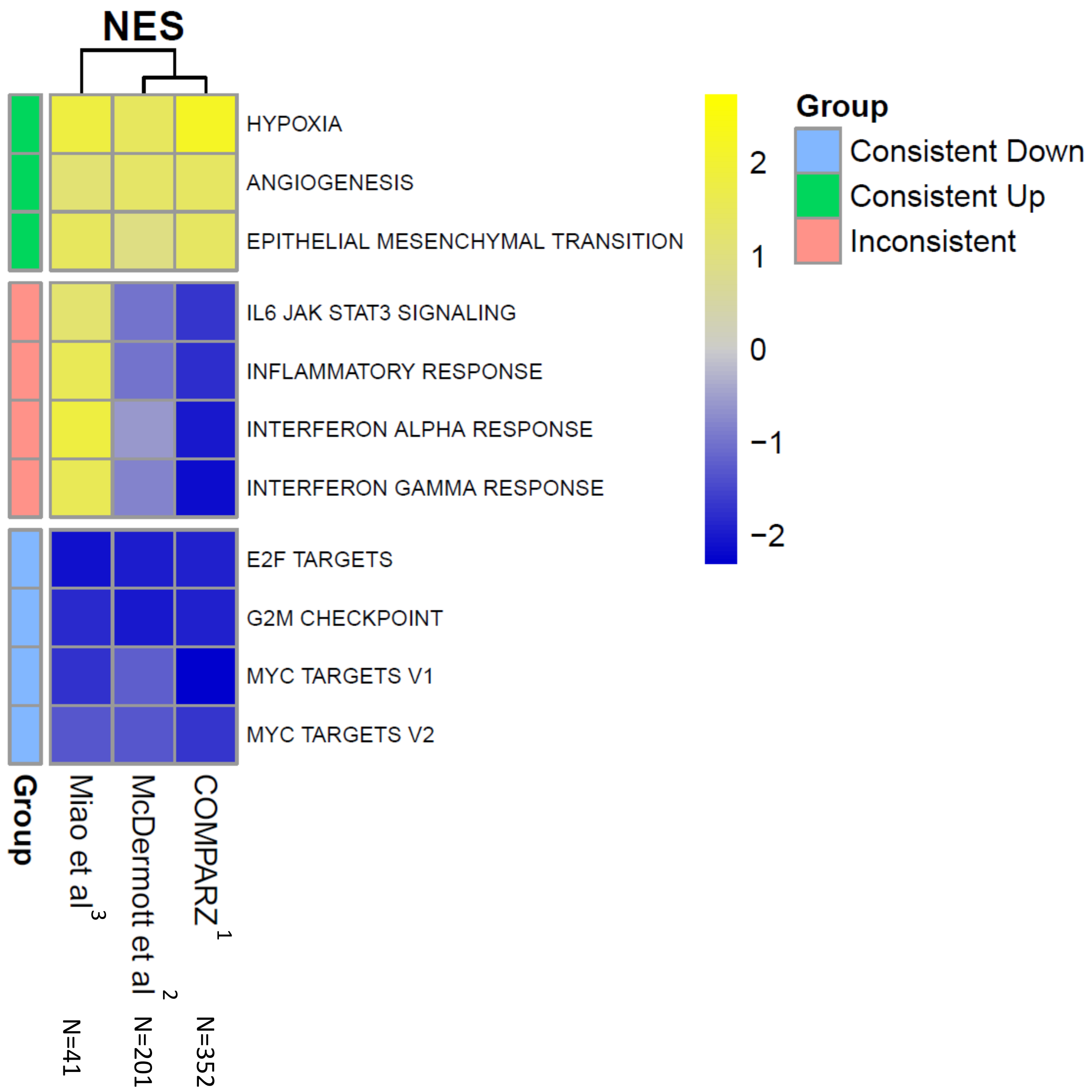

Supplementary Figure 4: Immune deconvolution of bulk express data. When stratified by *PBRM1* mutation status, no specific immune enrichment patterns were observed across the three cohorts (COMPARZ,[1] McDermott et al,[2] and Miao et al[3]).

Supplementary Table 1: Cox model demonstrating the association between overall survival and PBRM1 mutation stratified by cancer type. PBRM1 mutations were not significantly associated with overall survival in a cohort of 11 cancer types and remained insignificant after adjusting for TMB and total CNA. p-values derived from Cox proportional hazards model.

PBRM1 stratified by cancer type			
**N = 2,936**	HR	95% CI	p-value
**PBRM1 wild type**	Ref.		
**LOF**	0.92	0.62, 1.38	0.70
**Non-LOF**	1.03	0.73, 1.46	0.86
**PBRM1 stratified by cancer type (adjusted)**			
**N = 2,822**	HR	95% CI	p-value
**PBRM1 wild type**	Ref.		
**LOF**	1.21	0.80, 1.81	0.37
**Non-LOF**	1.32	0.92, 1.90	0.13
**TMB Score**	0.99	0.99, 0.99	<0.001
**Fraction CNA**	1.33	1.08, 1.64	0.008
**Drug class**			
**CTLA4 | PD-1/PD-L1**	Ref.		
**PD-1/PD-L1**	1.22	1.04, 1.42	0.013

Supplementary Table 2: Univariate and multivariate models of overall survival in *PBRM1* and/or *ARID2* mutated patients in MSKCC ICB cohort. *p*-values derived from Cox proportional hazards model.

PBRM1 OR ARID2 stratified by cancer type			
N = 2936	HR	95% CI	p-value
PBRM1 or *ARID2* LOF			
Wild-type	Ref.		
LOF	0.85	0.65, 1.12	0.25
Non-LOF	0.89	0.68, 1.15	0.36

ARID2 stratified by cancer type			
N = 2936	HR	95% CI	p-value
PBRM1 or *ARID2* LOF			
Wild-type	Ref.		
LOF	0.81	0.57, 1.15	0.24
Non-LOF	0.72	0.52, 1.00	0.052

ARID2 stratified by cancer type adjusted			
N = 2822	HR	95% CI	p-value
ARID2 LOF			
Wild-type	Ref.		
LOF	0.99	0.69, 1.43	0.95
Non-LOF	0.89	0.63, 1.25	0.50
TMB Score	0.99	0.99, 1.00	<0.001
Fraction CNA	1.33	1.08, 1.64	0.008
Drug Class			
CTLA4 | PD-1/PD-L1	Ref.		
PD-1/PD-L1	1.22	1.05, 1.43	0.010

PBRM1 or ARID2 stratified by cancer type adjusted			
N = 2822	HR	95% CI	p-value
PBRM1 or *ARID2* LOF			
Wild-type	Ref.		
LOF	1.09	0.82, 1.45	0.54
Non-LOF	1.10	0.84, 1.45	0.48
TMB Score	0.99	0.99, 0.99	<0.001
Fraction CNA	1.33	1.08, 1.64	0.008
Drug Class			
CTLA4 | PD-1/PD-L1	Ref.		
PD-1/PD-L1	1.22	1.04, 1.42	0.012

Supplementary Table 3: Multivariate model of *PBRM1* in non-small cell lung cancer. *p*-values derived from Cox proportional hazards model.

N = 983	HR	95% CI	p-value
PBRM1 LOF			
PBRM1 wild-type	—	—	
LOF	2.86	1.72, 4.74	<0.001
Non-LOF	1.78	0.88, 3.61	0.11
TMB Score	0.98	0.97, 0.99	<0.001
Genome doubled	1.21	0.89, 1.64	0.23
Fraction CNA	1.17	0.67, 2.04	0.58
Drug Class			
CTLA4	PD-1/PD-L1	—	—	
PD-1/PD-L1	1.30	1.00, 1.69	0.054

Supplementary References

1. Motzer, R.J., et al. Pazopanib versus sunitinib in metastatic renal-cell carcinoma. N. Engl. J. Med. 369, 722-731 (2013).

2. McDermott, D.F., et al. Clinical activity and molecular correlates of response to atezolizumab alone or in combination with bevacizumab versus sunitinib in renal cell carcinoma. Nat Med (2018).

3. Miao, D., et al. Genomic correlates of response to immune checkpoint blockade in microsatellite-stable solid tumors. Nat Genet 50, 1271-1281 (2018).
